# Quantitative Insights into the Adsorption Structure of Diindeno[1,2-*a*;1′,2′-*c*]fluorene-5,10,15-trione (Truxenone) on a Cu(111) Surface Using X-ray Standing Waves

**DOI:** 10.1021/acsomega.1c04799

**Published:** 2021-12-08

**Authors:** David A. Duncan, Philip J. Blowey, Tien-Lin Lee, Francesco Allegretti, Christian B. Nielsen, Luke A. Rochford

**Affiliations:** † Diamond Light Source, Harwell Science and Innovation Campus, Didcot OX11 0DE, U.K.; ‡ Physics Department, University of Warwick, Coventry CV4 7AL, U.K.; § Physics Department E20, Technical University of Munich, James Franck Straße 1, D-85748 Garching, Germany; ∥ Department of Chemistry, Queen Mary University of London, Mile End Road, London E1 4NS, U.K.; ⊥ Chemistry Department, University of Warwick, Coventry CV4 7AL, U.K.; # Chemistry Department, University of Birmingham, University Road, Birmingham B15 2TT, U.K.

## Abstract

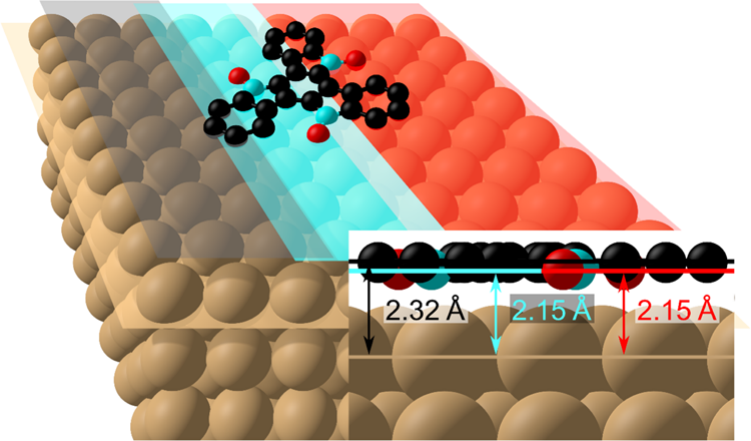

The adsorption structure of truxenone on Cu(111) was determined quantitatively using normal-incidence X-ray standing waves. The truxenone molecule was found to chemisorb on the surface, with all adsorption heights of the dominant species on the surface less than ∼2.5 Å. The phenyl backbone of the molecule adsorbs mostly parallel to the underlying surface, with an adsorption height of 2.32 ± 0.08 Å. The C atoms bound to the carbonyl groups are located closer to the surface at 2.15 ± 0.10 Å, a similar adsorption height to that of the chemisorbed O species; however, these O species were found to adsorb at two different adsorption heights, 1.96 ± 0.08 and 2.15 ± 0.06 Å, at a ratio of 1:2, suggesting that on average, one O atom per adsorbed truxenone molecule interacts more strongly with the surface. The adsorption geometry determined herein is an important benchmark for future theoretical calculations concerning both the interaction with solid surfaces and the electronic properties of a molecule with electron-accepting properties for applications in organic electronic devices.

## Introduction

Monolayers and sub-monolayers of electronically conjugated organic molecules are the compulsory first steps in building films and crystals for devices and applications. At these early stages of growth, a wide variety of structural polymorphs and bonding motifs can be observed even when only a single type of molecule is present. Changes in the bonding motif can affect how well molecules interact electronically with substrates^1^ and their thermal stability^2^ and can even modify their electronic properties.^3^ For these reasons, determining the structure at this stage of growth is of the utmost importance to understand how and why molecular semiconductors assemble. Quantifying the distance between the atoms comprising the adsorbate and the surface provides insights into the surface–molecule interactions and can provide a corollary to the wealth of studies present in the solution or solid-state coordination chemistry.^4,5^ When used as active materials in devices, organic semiconductors are often in direct contact with metal electrodes, for example, copper or gold.^6^ Understanding metal/organic interfaces can aid the design of more efficient devices and provide insights into the factors which determine (and limit) the performance.

Truxenone (diindeno[1,2-*a*;1′,2′-*c*]fluorene-5,10,15-trione) (shown schematically in Figure 2c) has been suggested as a replacement of fullerene electron acceptors in organic electronic devices.^7^ Fused heterocyclic molecules, such as truxenone, can be extensively chemically modified to tune their electronic structure, allowing a more efficient electron acceptor character.^8−11^ Addition of electronegative fluorine atoms to the perimeter of truxenone also influences its surface adsorption properties.^12^ Truxenone derivatives have also been used to construct covalent–organic frameworks for use as battery cathodes^13^ and create oligomeric semiconducting materials.^14^ The preceding examples show that truxenones are both interesting and useful as discrete molecules and as building blocks in organic and materials chemistry.

Here, we present synchrotron X-ray photoelectron spectroscopy (XPS) and a quantitative normal-incidence X-ray standing wave (NIXSW)^15^ measurement of the chemical and geometric structure of truxenone deposited on the Cu(111) surface. This molecule adsorbs at ambient temperature in a commensurate (8 × 8) structure on Cu(111)^16^ and represents a well-characterized system with, due to its commensalism, a well-defined rotational and translational symmetry with respect to the supporting surface.

## Results

**Figure 1:**
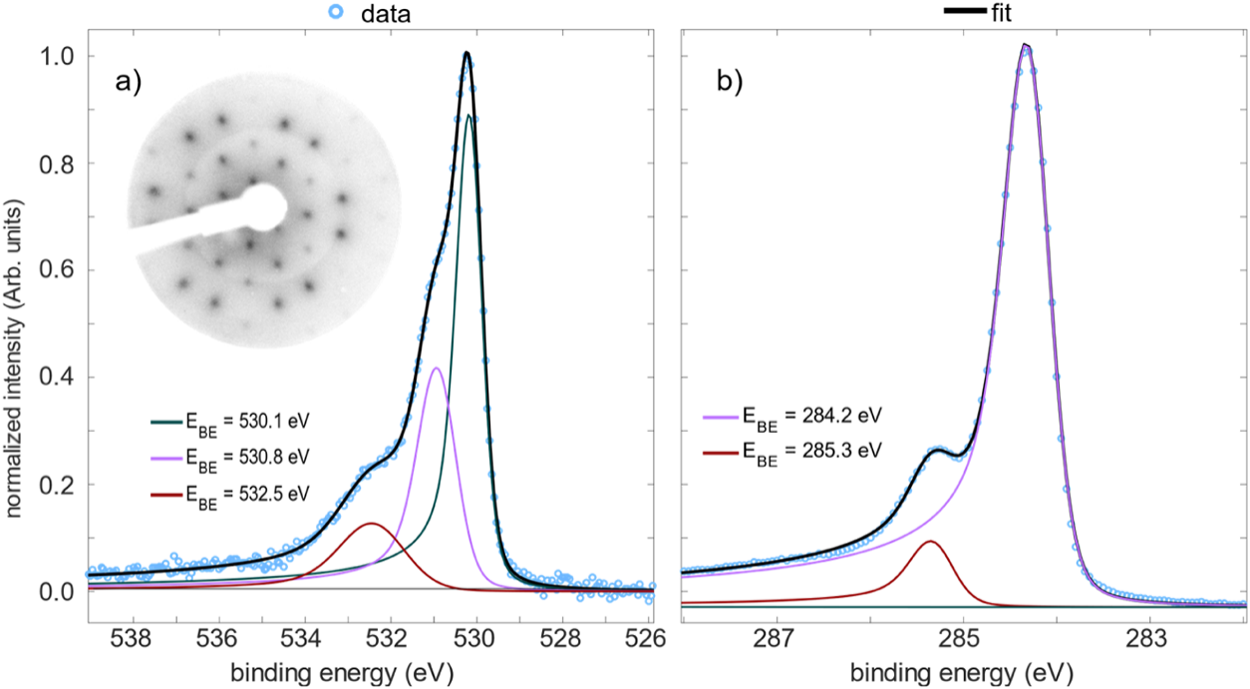
(a) O 1s (*h*ν = 2350 eV) and (b) C 1s (*h*ν = 641 eV) XPS spectra of Cu(111)/*p*-(8 × 8) truxenone; a LEED pattern (beam energy of 20 eV) of the same surface is given in the inset of (a).

The C 1s XPS spectra (Figure 1) consist of a primary peak with a binding energy, *E*_BE_, of 284.2 eV and a secondary feature at 285.3 eV with a ratio (integrated areas) of ∼9:1. The measured binding energy and observed peak ratio (compared to the nominal ratio of 24:3 of phenyl and ketone C atoms in the molecule) suggest that the 284.2 eV peak is related to the C atoms in phenyl rings and the 285.3 eV peak represents ketone C atoms. Such a binding energy, 285.3 eV, agrees well with the other C 1s spectra of C atoms in carbonyl or methoxy groups present in the literature (see Table 1). O 1s XPS spectra are somewhat more complicated, exhibiting three separate features at binding energies of 530.1, 530.8, and 532.5 eV (an integrated area ratio of 4:2:1). The origin of these three species is not obvious from the molecular structure of truxenone—all three O species would be expected to be chemically equivalent due to the structure and symmetry. These three peaks could, most simply, indicate three unique adsorption sites for the oxygen atoms at the Cu(111) surface. Despite the highest binding energy peak being significantly broader than the other two, we do not ascribe it to an energy loss feature due to the results of the NIXSW analysis (discussed later). One alternative possibility is that a small minority of truxenone molecules are present on the top of the ordered (8 × 8) islands, leading to a lower coherent fraction and higher apparent height (this is discussed further later in the text).

**Table 1:** Binding Energies and Adsorption Heights of the O 1s Components Found Here, Compared against Literature Values for Carbonyl, Alcohol, and Carboxylate Groups^b^

system	O species	O 1s BE (eV)	C 1s BE (eV)	O adsorption height (Å)
truxenone on Cu(111)		530.1/530.8	285.3	2.15/1.96
formate on Cu(111)	carboxylate	531.4^42^	287.5^42^	1.92–1.98^43, 44^
glycine on Cu(111)	carboxylate	531.6^45^	288.3^45^	1.98–2.00^46^
methanol on Cu(111) (deprotonated)	methoxy/carbonyl	530.9^47^	285.7^47^	
methanol on Cu(110) (deprotonated)	methoxy/carbonyl	530.8^48^	286.2^48^	1.22–1.47^48^
methanol multilayer (intact)	alcohol	533.1^49^	286.5^49^	
6,13-pentacenequinone	carbonyl	530.0^50^	285.1^50^	2.02^a^^50^
5,7,12,14-pentacenetetrone	carbonyl	530.1^50^	285.2^50^	1.98^a^^50^
uracil on Cu(111)	carbonyl	531.1–531.9^51^		
uracil on Cu(110)	carbonyl			1.83–1.90^52^
thymine on Cu(110)	carbonyl	531.1^53^		1.87–1.90^54^
cytosine on Cu(110)	carbonyl	531.1^53^		1.90^55^
5-fluorouracil on Cu(111)	carbonyl	530.9–532.2^56^	290.0–287.3^56^	
tetrahydroxybenzene on Cu(111) (intact)	alcohol	532.6^57^	285.3	
tetrahydroxybenzene on Cu(111) (deprotonated)	methoxy/carbonyl	530.8^57^		
diethylstilbestrol on Cu(111) (intact)	alcohol	532.7^58^	285.8	
diethylstilbestrol on Cu(111) (deprotonated)	methoxy/carbonyl	530.9^58^	285.8	
CuO	oxide	529.4^59^		
Cu_2_O	oxide	530.3^60^		

a Note that the associated coherent fractions for these species are 0.17 and 0.22; thus, it is unlikely that these O atoms sit at a single adsorption height.^17^

b Also given are the binding energy of the C 1s XPS spectral component corresponding to the C atoms that are bound to the given O species.

**Figure 2:**
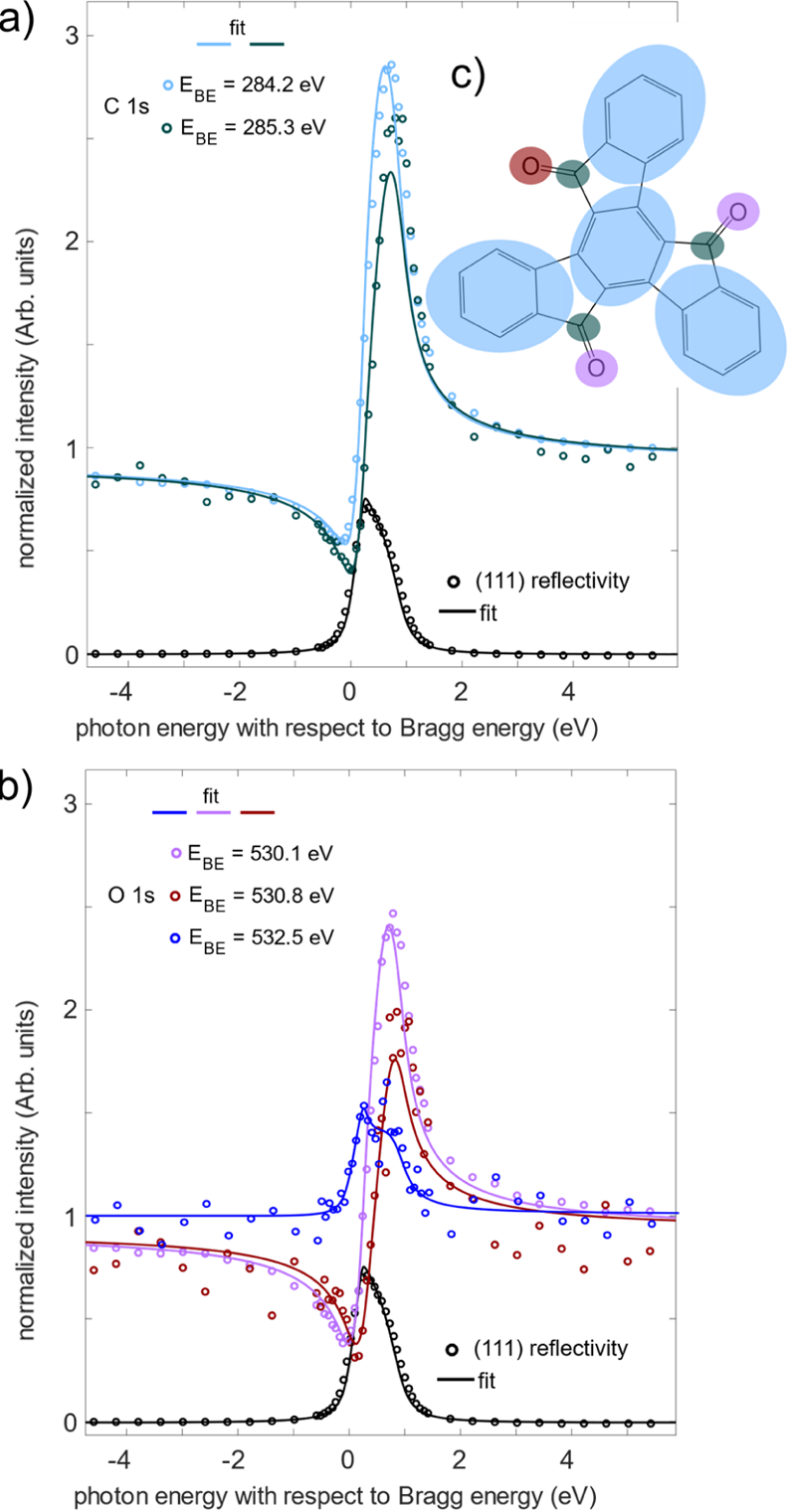
(a) (111) XSW profiles from both carbon species and (b) three oxygen species for Cu(111)/*p*-(8 × 8) truxenone. (c) Schematic of the truxenone molecule, with the different groups (phenyl carbon, ketone carbon, etc.) highlighted in the color related to their corresponding XSW profile.

**Figure 3:**
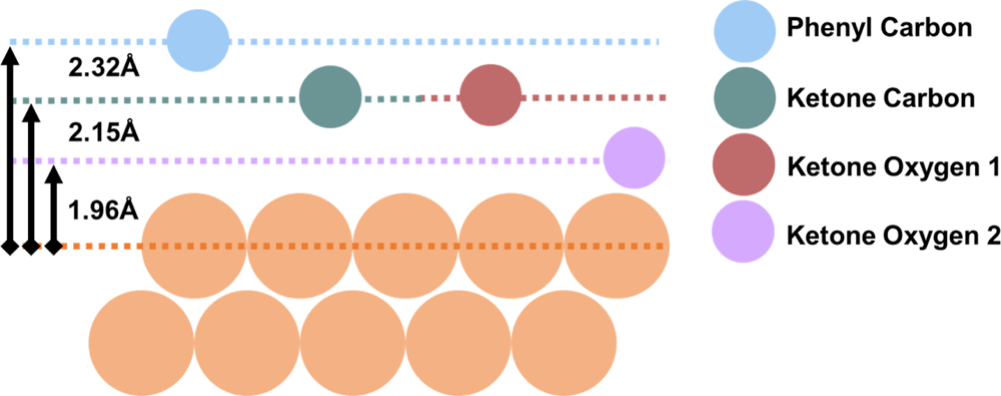
Schematic of the binding distances [displacement away from the Cu(111) surface] in the (8 × 8)truxenone/Cu(111) surface. The Cu(111) surface is indicated by the orange circles at the bottom of the image and arrows on the left (and labels) indicate the distances between atoms and the surface.

The individual energy distribution curves (EDCs) of the O 1s and C 1s NIXSW profiles were fitted assuming the same peak separations (in the binding energy) were present as those in the respective XPS spectra. Figure 2a shows the NIXSW profiles for the two carbon species, and Figure 2b shows the NIXSW profiles for the three oxygen species. Both carbon species and the two lowest binding energy oxygen species exhibit similar profiles, showing that they are all positioned at similar positions relative to the spacing of the wavefield generated by the Cu(111) crystal. In turn, this suggests that they are all positioned at a similar height above the surface. Coherent fractions and positions for these fits, as well as the inferred heights to which the latter corresponds if they lie below the first or second *d*_111_ layer spacing, are listed in Table 2, and the mean adsorption heights are shown pictorially in Figure 3. The phenyl C atoms (*E*_BE_ = 284.2 eV) are the most distant atoms to the nearest bulk lattice plane (most likely the surface, in the absence of relaxations) with an effective adsorption height of 2.32 ± 0.08 Å. C atoms originating from ketones effectively share the same adsorption height with the lowest-binding-energy O peak (*E*_BE_ = 530.1 eV), 2.15 ± 0.10 and 2.15 ± 0.06 Å (respectively), suggesting that this O species is the ketone oxygen atoms. The middle O species (*E*_BE_ = 530.8 eV) adsorbs closer to the surface (1.96 ± 0.08 Å).

**Table 2:** Coherent Fractions [*f*_111_], Coherent Positions [*p*_111_], and the Resulting Adsorption Heights in the First [*d*_111_(*p*_111_)] and Second [*d*_111_(1 + p_111_)] Cu(111) Layer Spacing above the Surface Termination^a^

	*f*_111_	*p*_111_	*d*_111_(*p*_111_)/Å	*d*_111_(1 + *p*_111_)/Å
C 1s *E*_BE_ = 284.2 eV	0.97 ± 0.10	0.11 ± 0.04	0.23 ± 0.08	2.32 ± 0.08
C 1s *E*_BE_ = 285.3 eV	0.91 ± 0.10	0.03 ± 0.05	0.06 ± 0.10	2.15 ± 0.10
O 1s *E*_BE_ = 530.1 eV	0.93 ± 0.08	0.03 ± 0.03	0.06 ± 0.06	2.15 ± 0.06
O 1s *E*_BE_ = 530.8 eV	0.88 ± 0.10	0.94 ± 0.05	1.96 ± 0.08	4.05 ± 0.08
O 1s *E*_BE_ = 532.5 eV	0.25 ± 0.06	0.83 ± 0.05	1.73 ± 0.10	3.82 ± 0.10

a Note that *d*_111_ is the (111) layer spacing of copper, 2.0871 Å.

The origin of this middle O species is unclear. The binding energy is indicative of a carbonyl species bound to Cu rather than an alcohol group (see Table 1), indicating that there has been no protonation of either of the low-binding-energy oxygen species (e.g., via surface-assisted tautomerization). Similarly, the adsorption height of this species is similar to that of the most previously studied carbonyl or carboxylate groups in the literature (see Table 1). It is notable that the peak shape of the middle-binding-energy species is somewhat broader than the lowest-binding-energy species, perhaps suggesting a less well-defined/more disordered adsorption site, though no significant difference is observed in the coherent fraction of the two species. Thus, we are led to conclude that the two O species both relate to carbonyl oxygen but in two local adsorption sites that are significantly different, enough to result in a measurable difference in binding energies. Two possibilities could explain this observation: a second whole-molecule truxenone (minority) adsorption site is present on the surface, or one of the three O atoms within the truxenone molecule binds to a different site with respect to the lateral structure of the Cu(111) surface. The latter model (where a single O atom per molecule is distinct) is shown schematically in Figure 2c. Note that the adsorption height of the lowest-binding-energy species is significantly greater than that seen for most O–Cu species (see Table 1). This may suggest that the lower-binding-energy species, either due to the flat conformation of the molecule or competing intermolecular interactions with the neighboring molecules, cannot adsorb in what would otherwise be the ideal O–Cu adsorption site and that the minority (middle binding energy) species is sitting in such a site.

The final O species (*E*_BE_ = 532.5 eV) exhibits a different coherent position from the other two O species, directly indicating that it is not an energy loss feature. This coherent position could indicate that the mean adsorption height of this species is either lower on the substrate (1.73 ± 0.10 Å) or significantly higher (3.82 ± 0.10 Å). However, its coherent fraction (0.25 ± 0.06) is dramatically lower than any other C or O species in this system (0.88–0.97), which indicates that this minority species likely occupies a large range (>0.5 Å) of adsorption heights,^17^ which in turn reasonably excludes the lower adsorption height on the surface. This may be due to the diffusion of molecules not incorporated into the (8 × 8) lattice or a second disordered layer of molecules atop the first. The possibility of charge transfer (from Cu to truxenone) breaking the C3 symmetry of the molecule and hence producing more than one surface–oxygen distance is recognized by the authors, but we have no experimental data to support this suggestion. Other organic semiconductors do undergo electronic symmetry reduction on Cu(111),^18,19^ and we will bear this point in mind in our future studies of this system.

## Discussion

The NIXSW results, collected and shown schematically in Figure 3, suggest that the molecule is adsorbed via the three ketones, at an adsorption height that would correspond to that of a chemisorbed species.^20−23^ The difference in height between the phenyl carbons and ketone carbons is 0.2 ± 0.1 Å, and this is spread across a molecule with a diameter of approximately 8 Å. Combined with the relatively high coherent fractions (0.88–0.97), this suggests that the truxenone molecule is mostly planar when adsorbed on Cu(111) (except for the bending of keto groups addressed below).

The 4:2 ratio between the two chemisorbed ketone oxygens (*E*_BE_ = 530.1 eV and *E*_BE_ = 530.8 eV) suggests that a large proportion of O atoms adsorb slightly closer to the surface. Either approximately one-third of the truxenone molecules have all three ketone O atoms in a lower (closer to the surface) adsorption site, implying two unique adsorption structures, that is, two wholly differently adsorbed “whole molecules”, or one O atom per molecule adsorbs at a lower adsorption height, implying two unique local adsorption sites for the O atoms. Extensive previous characterization of the (8 × 8) structure^16^ (confirmed by our experimental low-energy electron diffraction (LEED) pattern, shown as an inset in Figure 1a) shows that only a single type of supramolecular ordering is present. In particular, scanning tunneling microscopy (STM) images indicate that each (8 × 8) unit cell contains six “lobes” arranged in two mirrored triangular shapes that are slightly offset from one another and with an area of bare copper in between. These bright lobes likely relate to a phenyl ring in the truxenone molecule (the most electron-rich sub-molecular feature) and suggest two molecules per (8 × 8) unit cell. Furthermore, the distance between O atoms in the truxenone molecule (∼4 Å) is comparable to the Cu–Cu distance along the ⟨211⟩ directions (4.4 Å). Thus, were the vector between O atoms aligned with the ⟨211⟩ directions, all three O atoms could easily occupy identical adsorption sites. The natural assumption would be that all O atoms within an (8 × 8) unit cell would have the same adsorption height. Within our prior work on this system,^16^ high-density phases were observed on the surface, which could well have the O atoms in different adsorption sites. While our LEED results only indicated the presence of the (8 × 8) mesh, were any secondary phase present on the surface with an island size significantly smaller than the transfer width of a low-energy electron, it would not be observed in LEED patterns but would be observed in XPS/NIXSW results, though it is hard to believe that a phase could cover around one-third of the surface and not form islands large enough to be observed in LEED patterns. It is possible, therefore, that the O atoms of each molecule exhibit slightly different adsorption heights within the (8 × 8) mesh and that the vector between the O atoms do not align with a ⟨211⟩ direction. The presence of large quantities of a disordered phase is also unlikely as previous STM studies of truxenone^16,24^ and its fluorinated derivative^12^ show stable island formation at room temperature and sub-monolayer coverage.

The presence of a third species of O, which also exhibits a different coherent fraction and coherent position from the two lower-binding-energies species, suggests that there is a disordered/physisorbed species also adsorbed onto the surface. The area of this peak is around 1/7th of the overall O 1s area, suggesting that this species is very much in minority on the surface. No corresponding peak is observed in the C 1s spectra, and as the molecule seems to primarily interact with the substrate via the O atoms, it is perhaps not surprising that the C 1s spectra corresponding to this species would not exhibit a resolvable binding energy shift. It is also important to highlight that the coherent fraction of the C 1s spectra is quite high (0.91–0.97) compared to the expected coherent fraction for such a mixture of phases (∼0.8, assuming a 6:1 ratio). However, it is important to note that the uncertainty in the C 1s coherent fraction values is comparably large (±0.10), and thus, the disagreement is not as large as it would otherwise appear. Note that we cannot completely rule out the suggestion that this O species corresponds to a contaminant present in the evaporant that has far fewer C atoms per O atom, but we would highlight that the evaporant was triply purified by thermal gradient sublimation prior to deposition; thus, this seems unlikely.

We must also consider the pro-chiral nature of the truxenone molecule, which has been addressed in the previous studies of truxenones^25,26^ and similarly symmetrical molecules.^27−30^ Surface-induced enantiomers are clearly possible, but no “handedness” has ever been observed (using LEED or STM) in the (8 × 8) structure (or, for that matter, in the molecules themselves). This either means that they are indistinguishable (if they form islands with like-handedness molecules) or that the networks are racemic/insensitive to handedness. In any case, we do not see any physical reason why “R” or “S” molecules would interact with the Cu(111) surface in sufficiently different ways to affect a change in the binding energy of a core level or place the O atoms in an R or S molecule in a different coherent position. Were such a difference to exist, this could be the origin of the different adsorption heights.

## Conclusions

In this work, we have probed the chemical environment and quantitatively measured the structure of truxenone molecules on a Cu(111) surface using XPS and NIXSW. Three different O species were observed in XPS spectra at a binding energy of 530.1, 530.8, and 532.5 eV. All three species corresponded to different coherent positions in NIXSW profiles and thus are not energy loss features. The two lowest-binding-energy species are assigned to O atoms bound directly to the surface, but it is unclear if the two O species relate to O atoms within the same molecule at different adsorption heights or O atoms in different molecules. There are no data to support the presence of a significant secondary truxenone species, other than the (8 × 8) mesh. Density functional theory calculations could provide significant insights into this issue as the adsorption heights provided within this study would provide a stringent benchmark parameter for these calculations, and theoretically predicted O 1s binding energies for the O atoms in different local adsorption sites could well resolve the origin of these two O species. The ketone C atoms were coplanar with the majority O species, whereas the phenyl C atoms exhibited a slightly higher adsorption height (0.17 ± 0.13 Å). This difference in the adsorption height implies a small “bend” to allow the C of the ketone to approach the surface, but the small magnitude of the difference and the high coherent fractions (found throughout our study) indicate that there are no drastic deformations of the molecule. The comparatively small bond length of the molecule with the surface (1.96–2.32 Å) suggests chemisorption, which could result in significant hybridization of the molecular states of the molecule with the underlying surface. A similar behavior was previously observed for largely planar porphine molecules on the same surface,^31^ which adsorbed at a similar adsorption height (2.08–2.20 Å).^32^ Future studies of the electronic structure of truxenone, using either ultraviolet photoelectron spectroscopy or near-edge X-ray absorption fine structure, to (respectively) probe the occupied and unoccupied electronic states could be enlightening in this regard.

## Experimental Methods

Truxenone was synthesized from indane-1,3-dione according to a literature procedure.^33^ Measurements were performed at the I09 beamline^34^ at the Diamond Light Source (Oxfordshire, UK). A Cu(111) crystal (Surface Preparation Laboratory, NL) was cleaned by argon sputtering (1 kV) and annealing (725 K) with cleanliness and order confirmed by LEED and XPS. O 1s and C 1s XPS spectra were acquired at 2350 and 641 eV, respectively, and the absolute binding energy scale was set by the subsequent acquisition of a Cu 3p_3/2_ XPS spectrum (a binding energy of 75.2 eV^35−37^) at the same photon energy as that for both the O 1s and C 1s XP spectra. Truxenone, which had been triply purified by thermal gradient sublimation, was evaporated at 220 °C (measured by a K-type thermocouple) from an organic material effusion cell (Karl Eberl GmbH) onto a Cu(111) crystal held at ambient temperature. This resulted in the expected (8 × 8) LEED pattern (measured with multichannel-plate LEED, OCI Vacuum Microengineering Inc. and shown exemplarily in Figure 1a), indicative of the previously reported “porous” commensurate network.^24^ The XPS and NIXSW data were acquired using a VG Scienta EW4000 HAXPES hemispherical electron analyzer (acceptance angle ±28°) mounted with the center of its acceptance range perpendicular to the direction of the incident light, in the plane of the photon polarization (linear horizontal).

NIXSW measurements were acquired from the (111) Bragg reflection of Cu (*E*_Bragg_ ≈ 2972 eV) at near-normal incidence, and the photoelectron yield was monitored from O 1s and C 1s core levels. The corresponding layer spacing, *d*_111_, is 2.0871 Å. The reflectivity was monitored with a charge-couple device camera observing a fluorescent screen mounted on the port of the incident X-ray beam, simultaneously to the NIXSW measurements. Prior to each NIXSW scan, a (111) Bragg reflection was acquired. The reflectivity was fitted roughly with a Gaussian line shape, whose center was used to define the central photon energy for the NIXSW scan. NIXSW scans were then acquired in a photon energy window of ±5 eV around this central photon energy. Each NIXSW data set consisted of measured EDCs of C 1s and O 1s photoemission as a function of the photon energy using the analyzer in a fixed-energy mode (i.e., fixed pass energy and fixed retardation voltage, acquiring a range of kinetic energies in a single snapshot) and a pass energy of 500 eV. The measurements were acquired over 36 unique geometric positions (differing lateral positions of the beam on the sample) on the Cu(111) crystal (all close to the surface normal), resulting in 18 individual C 1s and O 1s NIXSW data sets. The summation of these 18 repeated scans of each core level were fitted using multiple peaks. Each peak was a convolution of a Gaussian line shape and a Doniach–Sunjic^38^ line shape. Over the NIXSW scan, the widths of the peaks were assumed to not vary and thus were fitted as a constant. As such, the intensity of these peaks was used to obtain the photoelectron yield modulated by the NIXSW effect. Variations in the photoelectron yield, due to the NIXSW effect, were modeled using the dynamical X-ray scattering theory.^39^ Two dimensionless fitting parameters, the coherent fraction, f_H_, and coherent position, p_H_, were obtained for each yield profile. The former parameter is related to the level of order in the system, the latter to the average position of the chemical species in question within the wavefield. Nondipolar effects in the angular dependence of the photoemission were accounted for with the asymmetry parameter Q,^15^ which was calculated theoretically.^40^ This calculation requires, as an input, the angle, θ,^41^ between the photon polarization and the emission angle. As the EW4000 HAXPES analyzer has a large acceptance angle (±28°), considering that the data were acquired at grazing emission (emission angles of 62–90°) and that the photoelectron emission rate varies significantly as a function of angle at grazing emission orientations, the mean angle of emission detected by the analyzer (weighted by the photoelectron intensity as a function of the emission angle) was used to calculate the *Q* parameter, as in the standard approach.^15^ This mean angle was determined by measuring an XPS spectrum at an off-Bragg photon energy and was determined to be 18°. Note that as the generated standing wave field has a period that matches the layer spacing of the substrate, the NIXSW technique directly determines where within that layer spacing the probed atomic species lies but not which layers it lies between. In the case of the (111) surface of Cu, where the layer spacing is close to 2 Å, NIXSW can easily differentiate between two species that differ in adsorption height by 0.1 Å but cannot differentiate between adsorption heights that differ by ∼2 Å. This is the so called modulo-*d* ambiguity––namely, the true adsorption height, *d*, is
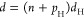
1where *d*_H_ is the corresponding layer spacing and *n* is an integer. Values of *n* greater than or equal to zero relate to adsorption above the surface, whereas values less than zero relate to absorption into the surface.
